# Antibody responses to surface antigens of *Plasmodium falciparum* gametocyte‐infected erythrocytes and their relation to gametocytaemia

**DOI:** 10.1111/pim.12323

**Published:** 2016-05-30

**Authors:** B. Dinko, E. King, G.A.T. Targett, C.J. Sutherland

**Affiliations:** ^1^ Department of Immunology and InfectionLondon School of Hygiene & Tropical MedicineLondonUK; ^2^ Department of Disease ControlLondon School of Hygiene & Tropical MedicineLondonUK; ^3^ Present address: Department of Biomedical SciencesSchool of Basic and Biomedical SciencesUniversity of Health and Allied SciencesHoGhana

**Keywords:** antibodies, antibody responses, gametocytes, *Plasmodium falciparum*, surface antigens

## Abstract

An essential element for continuing transmission of *Plasmodium falciparum* is the availability of mature gametocytes in human peripheral circulation for uptake by mosquitoes. Natural immune responses to circulating gametocytes may play a role in reducing transmission from humans to mosquitoes. Here, antibody recognition of the surface of mature intra‐erythrocytic gametocytes produced either by a laboratory‐adapted parasite, 3D7, or by a recent clinical isolate of Kenyan origin (HL1204), was evaluated longitudinally in a cohort of Ghanaian school children by flow cytometry. This showed that a proportion of children exhibited antibody responses that recognized gametocyte surface antigens on one or both parasite lines. A subset of the children maintained detectable anti‐gametocyte surface antigen (GSA) antibody levels during the 5 week study period. There was indicative evidence that children with anti‐GSA antibodies present at enrolment were less likely to have patent gametocytaemia at subsequent visits (odds ratio = 0·29, 95% CI 0·06–1·05; *P* = 0·034). Our data support the existence of antigens on the surface of gametocyte‐infected erythrocytes, but further studies are needed to confirm whether antibodies against them reduce gametocyte carriage. The identification of GSA would allow their evaluation as potential anti‐gametocyte vaccine candidates and/or biomarkers for gametocyte carriage.

## Introduction

Malaria is a major global health problem accounting for 198 million cases in 2014, and an estimated 584 000 deaths worldwide, 78% of which are thought to occur in African children under 5 years 1. Of the six parasite species causing malaria in humans, *Plasmodium falciparum* is the most virulent and the leading cause of morbidity and mortality among children under 5 2. For example in Ghana, malaria accounts for 30% of hospital admissions both in pregnant women and children under 5 years, and approximately 8% of these patients die every year 3. The development of resistance to antimalarials by malaria parasites and to insecticides by mosquitoes is increasing challenges 4. There is as yet no vaccine for malaria control, and targeting of multiple stages of the parasite may be required for any successful vaccine‐based strategy. As a result, there has been renewed interest in the sexual stages of the life cycle of malaria parasites, which involve distinctive parasite forms with specific morphology, metabolism and biochemical profiles needed to establish infection in the mosquito host 5, 6, 7. The sexual cycle begins with the development of gametocytes during human blood stage infection in all species, but a specific feature of *P. falciparum* is that only mature stage V gametocytes are seen in the peripheral circulation of infected individuals. The immature stages I to IV, representing the first 5–7 days of development, are instead sequestered in internal organs such as bone marrow and spleen 8, 9. It had been assumed that this is mediated by endothelium receptor−parasite ligand interactions, analogous to those seen in cytoadhesion of erythrocytes infected with mature asexual parasites 10, 11, but a puzzling lack of data to support this paradigm indicated that “…it remains possible that sequestration of immature gametocytes *in vivo* does not require the expression of adhesins on the erythrocyte surface” 12. Recent studies of changes in deformability of *P. falciparum* gametocyte‐infected erythrocytes during their development now show that the physical properties of stage I‐IV gametocytes, rather than adhesion to endothelium, are more likely the key to their pattern of retention in host tissues, and prevent emergence from sequestered niches into the circulation 13, 14.

Defined immune responses against variant antigens in asexual blood stage parasites have been described 15, 16. A major target of asexual stage immunity is the variant antigen family of PfEMP1, but other known targets include the Rifin 17, 18, STEVOR 19 and SURFIN antigen families 20, all of which may contribute to the surface antigen repertoire of asexual stage‐infected erythrocytes. The *var*,* rif* and *stevor* multi‐gene families coding for PfEMP1, Rifin and STEVOR proteins, respectively, are also known to be expressed in gametocytes and a role in the modification of the gametocyte‐infected erythrocyte surface remains a possibility 13, 21, 22. However, it has not been unequivocally demonstrated that any of these variants are surface‐exposed on the gametocyte‐infected host erythrocyte, or that they have a functional role in anti‐gametocyte immunity. In fact, there have been few studies on the natural immune responses to circulating gametocytes 23. In a study of plasma antibodies from Gambian children with a known history of gametocyte carriage and mosquito infectivity, we found some evidence that surface antigens, identity unknown, on erythrocytes harbouring mature gametocytes (GSA) of *P. falciparum* clone 3D7 were recognized by a subset of children 24. However, no evidence was found that the targets of these IgG responses were adhesins, as reactivity was only found to the most mature stage V gametocytes, which circulate in peripheral blood *in vivo*, and thus are not expected to express adhesins on the erythrocyte surface. Gametocyte surface antigens recognized by naturally occurring antibodies are potential vaccine candidates, as gametocyte clearance from the circulation would result in the interruption of malaria transmission 25.

To better understand antibody responses to *P. falciparum* GSA, we investigated the prevalence and development of such responses in a cohort of school children sampled over a 5‐week period. Antibodies were identified by recognition of cultured gametocytes from 3D7 and from *P. falciparum* clinical isolates collected in 2012. We addressed the following questions: whether natural plasma antibodies recognize GSA on diverse parasite isolates; whether GSA antibody levels are maintained longitudinally in individuals; whether carriage of GSA antibodies affects concurrent or subsequent gametocytaemia; and whether anti‐GSA antibodies could be detected in individuals without patent parasitaemia or gametocyte carriage.

## Materials and Methods

### Study population and plasma samples

Plasma samples were obtained from a longitudinal cohort study of asymptomatic school children in Pokukrom, in the Ahafo Ano South district of the Ashanti region, Ghana. This is an area of high malaria transmission with two seasonal rainfalls. The study population, environment, study design and sampling procedures have been described previously 26. Briefly, asymptomatic school children of Pokukrom Methodist primary between the ages of 5 and 12 years were screened for asexual malaria parasites in finger‐prick peripheral blood. For each sample, a rapid immunochromatographic point‐of‐care test (RDT) for antigenaemia was carried out (Malaria Pf rapid test, Shenyang LTH Technology Development Company, Beijing, China), blood smears were made for microscopy, and approximately 400ul of blood was collected into a microtainer for plasma separation. All children were clinically examined by a trained nurse to ensure that no child had symptoms suggestive of malaria. The medical history and other information of each child were recorded in a case report form. The slides were read at Kwame Nakrumah University for Science & Technology by experienced microscopists. At the second weekly visit of the study team to the participating school, all asymptomatic children with confirmed parasitaemia (sexual or asexual or both) at the time of visit 1 were enrolled. Inclusion criteria were: a recorded axillary body temperature of <37·5°C, no history of fever in the previous 48 h and microscopically confirmed parasitaemia caused by *P. falciparum* with or without the presence of other *Plasmodium* species. After blood sample collection, all enrollees were treated under observation with a standard regimen of dihydroartemisinin‐piperaquine, comprising three daily doses each of 1–3 tablets, depending on the weight and age, of P‐ALAXIN (Bliss GVS Pharma LTD, Mumbai, India), containing 40 mg dihydroartemisinin and 320 mg piperaquine phosphate per tablet. Enrolled children were followed up for repeat finger‐prick blood samples weekly for a further 3 weeks.

Plasma samples were also tested from microscopy‐confirmed parasite negative individuals and a semi‐immune adult individual in convalescence following a successfully treated episode of falciparum malaria 3 weeks prior to blood sampling. Negative control plasma samples were obtained from malaria‐free non‐immune European adult donors. The study protocols were approved by the Ghana Health Service ethics committee, (reference GHS‐ERC‐08/7/10), and the London School of Hygiene & Tropical Medicine ethics committee (reference 5775). In addition, individual and community consent were obtained before enrolment into the study. Approval of the study was also required and obtained from the Ghana Education Service directorate of the district.

### Parasite prevalence by microscopy

**Figure 1:**
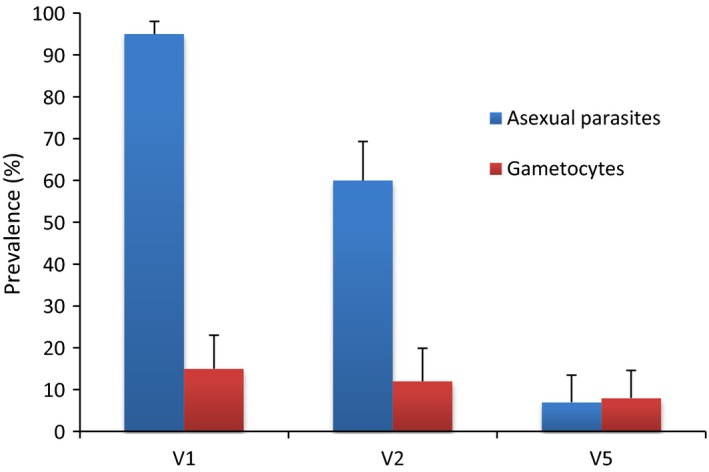
Asexual and sexual parasite prevalence in plasma donors for flow cytometry experiments. The baseline asexual and gametocyte burden of study participants (*N* = 113) whose plasma samples were tested in all flow cytometry experiments. Visit 1 (V1) samples were taken at the time of screening to identify parasitaemic children. Visit 2 (V2) samples were collected 1 week later, immediately before drug administration. Visit 5 (V5) samples were taken 3 weeks after treatment. A small proportion of children still harboured asexual parasitaemia at visit 5, 3 weeks after treatment (26). Error bars: upper 95% CI, estimated from the binomial distribution.

Asexual parasite and gametocyte carriage were determined by double‐read microscopy on thick and thin blood smears. Gametocyte density was determined by counting gametocytes against 500 leucocytes and asexual parasite densities were estimated from counts of asexual parasites against 200 leucocytes as described previously (Figure 1) 27.

### Gametocyte culture and purification

Gametocytes for antibody‐labelling experiments were produced from the 3D7 clone of *P. falciparum* and recent clinical isolates HL1204 and HL1205 28, according to established protocols 24, 29 with some modifications as follows. Freshly thawed 3D7 D‐sorbitol‐synchronized ring‐staged asexual parasites at 4% parasitaemia were used to initiate gametocyte production. Asexual blood stage parasites were cultured in human type O negative erythrocytes and incomplete RPMI 1640 medium (Sigma, UK) at a haematocrit of 3% with daily media changes. Before parasite cultivation, the medium was made complete by the addition of 5·96 g/L HEPES, 2 g/L sodium bicarbonate, 50 mg/L hypoxanthine, 3·96 g/L glucose, 0·003% l‐glutamine and 10% pooled blood type AB serum. Cultures were incubated at 37°C in a 3% CO_2_/1% O_2_/96% N_2_ gas phase according to established protocols 30. Parasite cultures were maintained through an additional asexual cycle, and sorbitol applied when ring‐stage trophozoites were abundant in Giemsa‐stained films. Gametocyte induction was then initiated by first introducing a proportion of spent medium to a tightly synchronized fast growing ring stage (day‐2) and with a subsequent increase in haematocrit from 3% to 5% when early to mid‐schizonts were seen (day‐1). Another round of sorbitol was applied 2 days later at which point haematocrit was decreased to 3% and the culture gave rise to young gametocytes, thus defining day 1 of gametocyte production. N‐acetyl glucosamine (NAG) was applied at a concentration of 55 mm throughout gametocyte culture to kill any remaining asexual stages 29, 31, 32.

**Figure 2:**
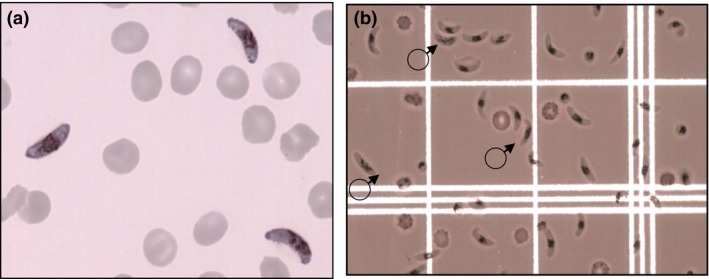
Purification of intact mature gametocytes. (a) Mature stage V gametocytes under high power oil immersion taken before parasite harvest and magnetic purification. The gametocytaemia observed in the sample shown was 16%. (b) Mature stage V gametocytes under low power objective on a haemocytometer after magnetic purification. The gametocytaemia observed is 75%, comprising mostly females. Three male gametocytes are indicated.

Mature gametocytes (14–15 days old) were harvested and purified for flow cytometry experiments with magnet‐activated cell‐sorting (MACS), 18 days after initiating asexual stage culture, as described 24, 33. Briefly, cultures containing mature stage V gametocytes were assessed for ex‐flagellation, washed in RPMI and applied to a magnetic cell‐sorting column with 21 G flow resistor at 37°C. Erythrocytes containing paramagnetic mature gametocytes were retained in the column while uninfected erythrocytes and debris passed through and were discarded. Parasites retained in the column were then released by removal of the magnetic field, washed, counted and aliquoted for antibody staining in preparation for flow cytometry. These were found overwhelmingly to comprise mature gametocytes within intact erythrocyte membranes (Figure 2), and the majority of these were female. Male gametocytes were observed at the expected prevalence of 10–20% (Figure 2b) 34.

For evaluation of antibody recognition of asexual parasite stages, mature schizonts and late trophozoites were harvested and purified for flow cytometry experiments by magnet‐activated cell‐sorting (MACS), as already described. Briefly, parasite cultures were washed in RPMI and applied to a magnetic cell‐sorting column with 21G flow resistor at 37°C. Erythrocytes containing mature parasites, which are paramagnetic, were retained in the column while uninfected erythrocytes and debris passed through and were discarded. Parasites retained in the column were then released by removal of the magnetic field, washed, counted and aliquoted for antibody staining in preparation for flow cytometry.

### Flow cytometry

Stage V gametocyte‐infected erythrocytes enriched by MACS purification were incubated with ethidium bromide (EB) at a final concentration of 0·1 mg/mL for 1 h, to enable discrimination of nucleic acid‐positive infected erythrocytes from uninfected anucleate erythrocytes. Aliquots of EB‐stained gametocytes (10^5^) were each incubated with test plasma samples for 30 min and then with goat anti‐human IgG antibody conjugated with Alexafluor488 diluted 1 : 500 for another 30 min. Stained parasites were washed three times with phosphate buffered saline (PBS) supplemented with 2% foetal calf serum (FCS) (PBS/2%FCS) after each antibody staining procedure. The EB‐ and antibody‐labelled parasites were then suspended in 300–500 μL of PBS/2%FCS and kept at +4°C overnight before flow cytometry analysis on the FACSCalibur (Becton Dickinson, UK).

**Figure 3:**
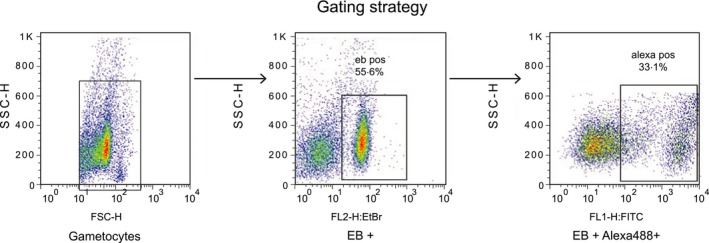
Gating strategy for flow cytometric analysis of antibody‐labelled gametocytes. The initial side scatter (SSC) and forward‐scatter (FSC) plots (left panel) obtained were gated out to identify the EB‐positive (infected) erythrocyte population (rectangle, middle panel). A double‐positive population of gametocytes binding to both EB and Alexfluor dyes is then derived (in square; right panel), comprising those gametocyte‐infected erythrocytes that are bound to antibody.

**Figure 4:**
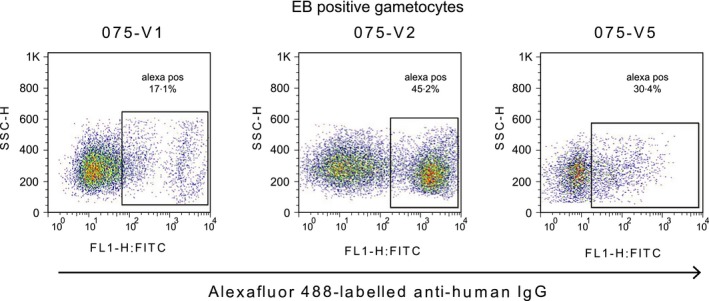
Plasma antibody recognition of mature gametocytes at visits 1, 2 and 5. Changes in antibody recognition relative to the first sampling time can be seen. The lower antibody levels recorded at visit 5 may be attributable to different gametocyte preparations used and not necessarily a reduction in antibody response.

Antibodies recognizing the surface of gametocyte‐infected erythrocytes were detected and measured by flow cytometric counting of IgG‐labelled, EB‐positive mature gametocytes according to established protocols for live cells as described 24, 35, 36. Before every experiment, compensation to exclude spectral overlap was carried out on samples singly labelled with EB or Alexafluor488. Data acquisition displayed samples in a bivariate plot of side scatter (SSC) vs. forward scatter (FSC), from which EB vs. side scatter plots were derived. A total of 10 000 events were acquired per sample. The EB‐positive events were gated against uninfected erythrocytes from the same donor source and used to further create bivariate plots of side scatter vs. Alexafluor488 to assist in exclusion of unstained erythrocytes, dead parasites, debris and non‐specific staining (Figure 3). A singly stained EB sample which was not stained for Alexafluor488 was used to set the Alexafluor488 positive gate position (Figure 4) for all samples collected during an experimental session. This fluorescence minus one control (FMO) was carried out each time data was acquired on the FACS Calibur.

For mature gametocytes, a total of four experiments were performed using different gametocyte preparations of 3D7. The first, second and third experimental sessions comprised 84 test samples from visit 1, 2 or 5, respectively, plus (as positive controls) five Gambian plasma samples from treated, symptomatic children who were microscopically confirmed gametocyte‐carriers, and characterizsed previously as strong gametocyte‐recognizers^24^. We also included five negative controls from European donors who had no known history of malaria. Our positive control plasma (known to strongly recognize gametocytes) were also tested against the donor erythrocytes used in each experiment, to rule out non‐specific recognition of the donor cell surface. The order of plasma selection was based on sample positions within a series of 96‐well plates in which working aliquots were kept. For the first 84 samples per each visit was any seven plasma samples per row of nine samples across 13 rows. A fourth experiment included all the remaining test plasma from the three visits (*N* = 29 for each of Visit 1, Visit 2 and Visit 5) in one session using a single gametocyte preparation (Table 1). Each experiment was performed once. Similar procedures were performed using developing and mature gametocytes of HL1204. Twenty‐five plasma samples comprising 20 from visit 1 and 5 from visit 5, characterized in the previous experiments with 3D7, were analysed in the HL1204 assay. The basis for selecting these 25 samples was the levels of antibody reactivity observed from 3D7 data. The 20 Visit 1 samples represented samples drawn from five highly reactive, 10 moderately reactive and five weakly reactive by our method of analyses. The Visit 5 samples were from the same individuals as the five highly reactive Visit 1 samples.

**Table 1:** Flow cytometry assays with mature 3D7 gametocytes and relationship to sampling visits showing the number and source of samples examined in each of the four experiments

Experiment^a^	Ghanaian sampling visits			Gambian controls	European controls	Total samples
	Visit1	Visit 2	Visit 5			
1	84	0	0	5	5	94
2	0	84	0	5	5	94
3	0	0	84	5	5	94
4	29	29	29	5	5	97

a Each experiment was performed with a different preparation of day 14 mature gametocytes of 3D7. Ex‐flagellation was observed in the preparations for experiments 1 and 2, but not for experiments 3 and 4.

### Design of analytical methods and definition of antibody binding

Flow cytometry data were analysed using flowjo software 37. Antibody binding was measured first as the percentage of EB‐positive cells also labelled by Alexafluor488 (double‐positives: DP). Secondly, geometric mean fluorescence intensity (MFI) of the double‐labelled cell sub‐population was estimated for each sample, at each visit and parasite stage. The %DP and MFI estimates for each category were dichotomized using the median as an assumption‐free, transparent cut‐off to define strong and weak antibody binding responses. This unconventional descriptive approach was deployed to compensate for the absence of established cut‐offs and gold standard analytical methods. Both parameters were further ranked in the order of strong to weak antibody responses.

### Data and statistical analyses

The proportion of gametocytes labelled by each plasma sample, %DP, was retained as a continuous variable, and also transformed into a binary variable using the median value as a cut‐off. We also analysed %DP using a simple ranking approach. The intensity of gametocyte recognition among the double‐labelled cells (i.e. MFI) was analysed similarly, as a continuous variable, a binary variable around the median, and by simple ranking. Age in years was considered as a continuous variable. Chi‐square tests were used to test for statistically significant differences between groups in the case of dichotomous variables while Wilcoxon rank sum tests were used to test for significant differences between groups in the case of continuous variables. Estimation of summary statistics and all tests of associations were performed in STATA software (Stata 12.0, Statacorp, Texas, US). Our analyses were based on *a priori* hypothesis‐driven questions generated from previous work 24. These questions were formulated and stated in the study protocol submitted for IRB approval prior to field work, and an analysis plan, drawn up before any statistical tests were performed, was followed (Dinko B. 2013. PhD Thesis, University of London). For these reasons, we did not apply Bonferroni's correction to account for random associations due to multiple comparisons.

## Results

### Longitudinal recognition of gametocyte‐infected red blood cells of *Plasmodium falciparum*

**Figure 5:**
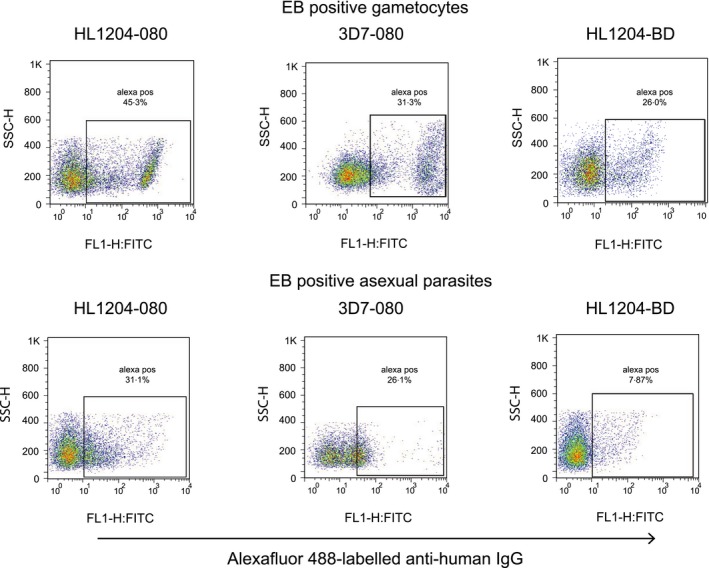
Comparison of antibody responses to 3D7a and HL1204. Antibody reactivity to purified Stage V gametocytes (above) and asexual stages (below) from both the 3D7 laboratory clone and the recently adapted polyclonal clinical isolate HL1204 (28). Study participant 080 (plasma from Visit 1) strongly recognized both gametocytes and asexual parasites from both lines. Convalescent plasma from research team member BD, a Ghanaian adult treated for symptomatic malaria at the conclusion of the fieldwork, recognized gametocytes of the Kenyan HL1204 parasites more strongly than asexual parasites of the same isolate.

We tested immune plasma from 113 asymptomatic Ghanaian school children with microscopy‐confirmed *P. falciparum* infection, representing the “per protocol” cohort from our study, having been seen at all five visits by the study team. Antibody recognition of the surface of gametocyte‐infected erythrocytes was tested by flow cytometry at 3 of the 5 consecutive weekly time points: Visit 1 (day ‐7; i.e. day of the first blood film screen to identify parasitaemic individuals), Visit 2 (day 0; i.e. when all bloodfilm‐positive children from Visit 1 received treatment) and Visit 5 (day 21 i.e. 4 weeks after screening, and 3 weeks after treatment). The microscopic prevalence of asexual and sexual stage *P. falciparum* in these children at each visit is shown in Figure 1 26. A reduction in asexual parasite prevalence between visit 1 and visit 2 could be attributed to immunity but not treatment, as the visit 2 sample was taken before drug administration. Children in this cohort harboured plasma antibodies which recognized the surface of erythrocytes infected with asexual parasites, (Figure 5; Tables 2 and 3; B. Dinko, PhD Thesis, University of London, 2013) as expected 24. Children also harboured IgG which recognized erythrocytes infected with mature gametocytes, but the level of recognition varied among individuals, and among the three time points in some individuals (Figure 4; Tables 2 and 3). Experiments 1, 2 and 3 evaluated plasma from time points 1, 2 and 5, respectively, for the first 84 participants. Each of the three experiments was performed with a different preparation of mature gametocytes. Plasma from all three time points for the remaining 29 individuals were analysed together in experiment 4, utilizing a single batch of gametocytes (Table 1). Paired analysis of results compared Visits 2 (day of treatment) and 5 for both proportion of labelled gametocytes (%DP) and mean fluorescent intensity (MFI) for plasma antibody from these 29 individuals. In 23 of these, MFI was higher at Visit 2 than at visit 5, indicating a significant reduction in labelling intensity over this 3 week period (*P* = 0·005; 2‐sided sign test). In contrast, only 11 of these individuals showed a reduction in %DP estimate at Visit 5 (*P* = 0·200). Thus, a similar proportion of gametocytes was labelled by plasma at the two time points, but the intensity of recognition appeared to have reduced over the 3 week interval between them. In contrast, 62 of the 84 Visit 5 plasma tested in experiment 3, in which ex‐flagellation of gametocytes was not seen, identified a lower proportion of labelled gametocytes than had been seen with the Visit 2 sample from the same individual in experiment 2, in which ex‐flagellation was successful (Table 1) (*P* < 0·0001). One explanation is that the maturity of gametocytes differed, and this affected Ab recognition in these two experiments.

**Table 2:** The proportions of parasites recognized by human IgG (%DP) and the intensity of recognition (MFI) of 3D7 gametocytes and asexual stages, showing the median and the range for each parasite stage and sample collection time point

Plasma collection time point	Gametocytes		Asexual stages	
	%DP Median (range)	MFI Median (range)	%DP Median (range)	MFI Median (range)
Visit 1 (*N* = 113)	13 (1–80)	199 (52–2493)	15 (4–74)	22 (18–143)
Visit 2 (*N* = 113)	28 (3–95)	56 (40–101)	7 (2–74)	48 (38–112)
Visit 5 (*N* = 113)	27 (9–53)	47 (38–78)	9 (4–47)	294 (39–1739)

**Table 3:** The proportions of parasites recognized by human IgG (%DP) and the intensity of recognition (MFI) of HL1204 gametocytes and asexual stages, showing the median and the range for each parasite stage and sample collection time point

Plasma collection time point (*N*)	Gametocytes		Asexual stages	
	%DP Median (range)	MFI Median (range)	%DP Median (range)	MFI Median (range)
Visit 1 (*N* = 16)	18 (13–36)	35 (23–62)	26 (20–71)	34 (20–248)
Visit 5 (*N* = 4)	22 (21–52)	33 (25–90)	18 (16–20)	36 (32–37)

In order to distinguish whether antibody responses to gametocyte‐infected erythrocytes also recognized asexual stages, we tested for associations between recognition of the two parasite stages at each sampling time. There was no association between the occurrence of antibody recognizing the surface of erythrocytes infected with asexual parasites and gametocyte‐infected erythrocytes at any time point (Table 4). Further, there was no difference in the likelihood of carrying anti‐GSA antibodies between gametocyte‐carriers and those with only asexual parasites (by microscopy) at Visit 1 (odds ratio (OR) 1·56, 95% CI: 0·48–5·25; *P* = 0·4).

**Table 4:** Lack of association between frequency of antibody recognition of the surface of erythrocytes infected with 3D7 gametocytes and of erythrocytes infected with asexual stages

Plasma collection time point	Odds ratio^a^	95% CI	*P*
Visit 1 (*N* = 113)	0·67	0·30–1·51	0·29
Visit 2 (*N* = 113)	0·60	0·27–1·36	0·18
Visit 5 (*N* = 113)	1·19	0·48–2·93	0·67

a Odds of the proportion of asexual parasites recognized being above the median if the proportion of gametocytes recognized is above the median.

### Ghanaian plasma antibodies recognize *Plasmodium falciparum* GSA expressed by a Kenyan isolate

To test directly whether the immune recognition observed in our previous work with 3D7 24, a laboratory‐adapted parasite line from a Dutch case of airport malaria first described in 1981 28, also occurs with currently circulating African parasites, we further tested plasma against mature gametocytes produced from HL1204, a clinical isolate obtained in 2012 from a UK falciparum malaria patient who had travelled to Kenya 28. Comparable frequency and intensity of antibody recognition of the gametocyte surface were observed with HL1204 as with the 3D7 clone (Figure 5). Importantly, these observations showed that gametocytes of East African origin can be recognized by antibodies from West African individuals. Convalescent plasma from Ghanaian adult BD, recently treated for clinical malaria caused by *P. falciparum* infection contracted in the study area, also exhibited strong antibody recognition of HL1204 gametocytes but only weakly recognized asexual parasites (Figure 5). For 14 plasma, we had sufficient data to evaluate associations between recognition of 3D7 gametocytes, and those of HL1204. Median %DP was significantly higher for the 3D7 gametocytes (43·75%, IQR 19·7–54·9%) than for HL1204 (7·08%, IQR 6·56–7·76%), and this difference also held in paired analysis for each plasma sample (signrank test *P* = 0·0010). MFI, in contrast, was greater for the HL1204 gametocytes (median 59·7%, IQR 46·1–75·7%) than for 3D7 (43·35%, IQR 37·5–47·4%), and this was significant in paired analysis (signrank test *P* = 0·012). This suggests that GSA expression occurred on fewer of our HL1204 gametocytes, despite recognition being more intense than on GSA‐positive 3D7, consistent with there being different proportions of mature, GSA‐expressing gametocytes in the two cultured gametocyte preparations, but more antibody bound per gametocyte in the HL1204 line. Among the 14 evaluable plasma samples, neither prevalence nor intensity of recognition of 3D7 gametocytes by a particular individual at a particular time point was associated with higher prevalence or intensity, respectively, of recognition of HL1204 gametocytes. Nevertheless, when analysed by %DP, four of the 14 immune plasma showed stronger recognition of HL1204 gametocytes compared to 3D7 gametocytes (Figure 5) and five plasma showed stronger responses to 3D7 gametocytes compared to HL1204. Five of these plasma samples exhibited strong recognition to gametocytes of both parasite lines, by the proportion of gametocyte recognized (%DP). Similarly, using the intensity of recognition (MFI), four plasma samples showed strong antibody binding intensity to gametocytes of both parasite lines and the remaining 10 samples recorded intensities that were stronger for gametocytes of HL1204 than 3D7 gametocytes.

### No detectable antibody binding to the surface of immature gametocytes of a Kenyan isolate

To test for any immune recognition of the surface of erythrocytes infected with immature gametocytes, 25 immune plasma known to recognize GSA were evaluated for labelling of erythrocytes infected with developing gametocytes of HL1204. This preparation was only partially synchronized, and microscopically determined to comprise 50% Stage IV, 25% Stage III and 25% Stage IIa/IIb gametocytes. As previously shown for 3D7 24, detectable levels of antibodies recognizing the surface of erythrocytes infected with immature gametocytes of *P. falciparum* were not observed.

### Plasma antibody responses in parasite negative individuals

In order to estimate the prevalence of anti‐GSA antibodies in the population as a whole, we tested plasma samples from microscopy‐confirmed parasite negative children not included in the longitudinal study but from the same school cohort. Twenty‐four of the 50 available samples (48%) recognized the surface of gametocyte‐infected erythrocytes from HL1204 after flow cytometry analysis. Thirty‐five individuals also harboured antibodies to the surface of asexual parasite‐infected erythrocytes. Among the 35 samples showing recognition to asexual parasite ‐infected erythrocytes, 12 had been previously shown to harbour sub‐microscopic parasitaemia by PCR 26.

### Anti‐GSA antibody recognition and gametocyte carriage

To determine whether any relationship could be discerned between immune responses raised against the surface of gametocyte‐infected erythrocytes and continuing carriage of gametocytes, we tested for associations between the presence of detectable anti‐GSA antibodies and carriage of gametocytes over the longitudinal sampling period. No association was found between antibody responses to the surface of gametocyte‐infected erythrocytes and concurrent carriage of gametocytes at any given time point (OR 1·56, 95% CI: 0·48–5·25; *P* = 0·4). Gametocytes were detected during follow‐up in 16 (17·2%) of the 93 individuals with no detectable gametocytes at visit 1. In 10 of these individuals, this occurred at visit 2, in the blood sample taken immediately prior to DP treatment. In one individual, this occurred at visit 4, and in the remaining five children gametocytes were microscopically detected for the first time at visit 5. There was a weak association between GSA antibody carriage at enrolment (%DP > median) and reduced likelihood of the appearance of gametocytes in subsequent visits (OR 0·29, 95% CI: 0·06–1·05; *P* = 0·03).

### Prevalence vs. Intensity of antibody recognition and relation to age

The mean fluorescence intensity among double‐labelled erythrocytes in our flow cytometry plots represents the intensity of antibody binding to the surface of gametocyte‐infected erythrocytes, while %DP denotes the proportion of gametocytes recognized by anti‐GSA antibodies. These are distinct measures, as plasma from some children showed strong antibody recognition measured by MFI while only recognizing a small percentage of cells, and *vice versa*. These two measures were strongly associated in our dataset at visit 1 (OR 8·40, 3·35–21·4), but not at visit 5 (OR 0·566, 0·251–1·27), suggesting fluctuations in the quantity and quality of anti‐GSA responses in this group of children over time, although differences in maturity of gametocyte preparations in the different experiments can only be excluded in Expt 4 (Table 1). As immune responses to gametocytes and gametes have been shown to be age‐dependent 38, we investigated the impact of age on GSA antibody prevalence. There was weak evidence that children older than the median age of 10 years (range 11–17, *N* = 52) were more likely, at visit 1, to already harbour antibodies that recognized the gametocyte surface with a high mean fluorescence intensity than were children 10 years or younger (range 5–10, *N* = 59; OR 2·15, 95% CI 0·943–4·94; *P* = 0·046). This weak relationship was also seen, though non‐significant, when %DP was considered (OR 2·08, 95% CI 0·91–4·80; *P* = 0·058).

## Discussion

We have shown that Ghanaian school children with asymptomatic malaria carry antibodies that recognize antigens, GSA, on the surface of *in vitro*‐cultured erythrocytes infected with *P. falciparum* gametocytes from two different parasite lines. In contrast, plasma antibodies did not recognize immature gametocyte‐infected erythrocytes from either the laboratory‐adapted parasite line 3D7, or from the recent Kenyan isolate HL1204. Anti‐GSA antibodies were maintained over the study period (5 weeks) in some individuals, and were detected among microscopically confirmed parasite negative children, and in a semi‐immune adult convalescing from symptomatic malaria. Thus in this high transmission area, parasitological status is not a good indicator of likelihood of carriage of antibodies against sexual or asexual *P. falciparum* parasites.

This study, lasting only a few weeks, does not provide an opportunity to assess whether these are long‐lived antibody responses, and the relation between human anti‐GSA antibody responses and recent exposure to gametocytes remains to be fully determined. Also, although we found some evidence that carriage of anti‐GSA antibodies is associated with reduced likelihood of the subsequent appearance of microscopically detectable gametocytes, this can only at present be a tentative conclusion subject to many variables.

Anti‐GSA antibodies were detected in microscopically gametocyte‐negative individuals, but this might be a consequence of sub‐microscopic gametocytaemia 38. Under high transmission, as in our study site, antibodies cannot be matched to any particular episode of detectable parasitaemia, as infectious mosquito bites can deliver new parasite inoculations regularly. Thus anti‐GSA antibody may remain circulating in the (apparent) absence of antigens, in contrast with the antibody responses to (non‐surface exposed) gamete antigens such as Pfs230 and Pfs48/45, which are short‐lived and thought to reflect recent exposure to gametocytes 38, 39, 40, 41. Cohort studies of anti‐GSA antibody carriage deploying exhaustive RNA‐based detection of circulating gametocytes, using QT‐NASBA or qRT‐PCR, are needed to fully elucidate this relationship.

We found strong antibody responses in all three time points tested and, in a small proportion of children, these were maintained over the study period. In others, fluctuating antibody responses were seen, and accurate measurement and comparison of antibody responses at single time points can thus be misleading. These findings therefore raise questions regarding how to set cut‐offs for defining antibody responses in a population where there is constant exposure to infection. There is also the technical difficulty, when deploying large volume, long‐term resource‐intensive gametocyte cultures, of ensuring the preparations are uniform in terms of gametocyte maturity in independent experiments; for example, we found some evidence that, when ex‐flagellation was not observed (in experiment 3), a lower proportion of gametocyte‐infected erythrocytes were recognized by IgG, perhaps due to the presence of late Stage IV/early Stage V gametocytes not yet presenting the GSA of interest.

There was unexpected poor antibody recognition at visit 5 which requires further investigation to establish whether this was a reproducible change between V2 and V5, or attributable to different gametocyte preparations used. Thus, in cultured gametocytes at least, the appearance of GSA may be transient and heterogeneous, and hence difficult to capture in a synchronous way. Repeat testing of a group of samples across all four experiments would have allowed us to address some of these questions. GSA expression patterns might also be dimorphic, differing between male and female mature gametocytes. Technical improvements in both gametocyte preparation and synchronization, as well as reliable sex discrimination within a more sophisticated multiplex‐labelling of parasites for flow cytometry would greatly assist in resolving these difficulties.

The procedure employed sought to prepare very mature gametocytes for the flow cytometry experiments, while minimizing the release of female gametes that might be enumerated as positive due to antibody recognition of the gamete surface, rather than that of the host erythrocyte. Our experimental design sought to minimize this risk, and checks were put in place: gametocytes were handled at 37°C, and then immediately chilled on ice prior to flow cytometry to minimize ex‐flagellation and female gamete emergence, side‐ and forward‐scatter parameters on the cytometer were set to count only cells with the size and shape of an erythrocyte, and Giemsa‐stained examination of preparations before (Figure 2) and after (not shown) flow cytometry were examined to verify that erythrocyte membranes are intact. While the majority (98%) of labelled cells were apparently intact erythrocytes harbouring gametocytes, a few emergent female gametes, known to express antigens recognized by naturally occurring antibody responses 40, 41, may have slipped though the gating strategy and contributed to our antibody signal. Future studies could utilize post‐flow cytometry immunofluorescence staining using gamete markers 21, 42 to determine the number of emergent gametes in the gametocyte preparations, or multiplex approaches in which a female gamete surface label is included, permitting isolation of these events from the analysis. Male gametes will not contribute to this signal as they will not be counted following side‐ and forward‐scatter filtering.

The identity (or identities) of the detected gametocyte surface antigens is not yet known. The recognition by Ghanaian plasma antibodies of two different parasite lines isolated from different countries and over 30 years apart indicates that GSA, or at least some of the component antigens, have a degree of conservation across geographical regions and isolates, as has been shown for transmission blocking vaccine candidate Pvs230 43. Similar findings have been observed in studies of variant surface antigens in asexual parasites where plasma antibodies recognized and/or agglutinated parasite isolates regardless of the geographical origin of the infected erythrocytes 44, 45, showing that components of parasite antigens may be conserved across different populations. Our sample size (*N* = 14) in the analysis of cross‐reactivity in GSA recognition between the two parasite lines was insufficient to adequately explore this question of the level of antigen conservation, and there was a lower prevalence of GSA‐expressing gametocytes, albeit with high intensity labelling, in the HL1204 line. Further work is needed with larger samples, from a wider variety of parasite lines. In addition, no association was observed between antibody recognition to gametocyte surface by the two parasite lines. This was possibly due to our methodological design where 3D7 gametocyte maturation protocols were used for HL1204. In future work, specific maturation protocols for HL1204 gametocytes may need to be developed.

We found no antibody binding to the surface of erythrocytes infected with unsynchronized immature gametocytes (stages II, III and IV) from the Kenyan patient isolate. This is consistent with an earlier finding 24 when testing immature gametocytes from 3D7a, and is in line with recent adhesion studies 14, 46 which conclude that developing gametocytes do not, as previously thought, sequester from the peripheral blood through adhesion to human bone marrow‐derived endothelial surfaces and receptors 9, 10, 11. Although we provide no evidence that developing gametocytes express surface antigens that may be involved in sequestration through parasite ligand‐host receptor interactions, we cannot rule this out; further studies with tightly synchronized preparations of immature gametocytes are required to further explore this question.

Antibody recognition of the surface of mature gametocyte‐infected erythrocytes was found to be more common (average prevalence 26% DP) than recognition of asexual parasite‐infected erythrocytes (average prevalence 6% DP) in plasma taken 3 weeks after cure with ACT when tested at the same time by flow cytometry. One possible explanation for the low asexual antibody recognition is a rapid decline in antibody titre to the dominant variant surface antigens, PfEMP1 25, as seen with anti‐merozoite antigens in the absence of infection 38, 47, although it may also reflect low surface expression levels of PfEMP1 in our asexual cultures. This decline may also be occurring during asymptomatic carriage in the cohort described here. It is known that gametocytes, unlike asexual stages, which are cleared by efficacious medication, continue to develop and emerge out of sequestration into circulation weeks after malaria treatment 48, 49, and may elicit further immune responses. We found no association between presence of GSA antibodies at enrolment and carriage of gametocytes at enrolment or during follow‐up. However, as mentioned above, we did find a weak, but significant reduction in the likelihood of appearance, emergence or development of gametocytes later in follow‐up among children with anti‐GSA antibodies at visit 1. There was also a significant association between carriage of GSA antibodies at visit 1 and 2 but not at visit 1 and 5, perhaps suggestive of some decay following treatment.

Our findings support the existence of antigens on the surface of a sub‐population of mature gametocyte‐infected erythrocytes which induce human plasma antibody responses. PfEMP1 proteins encoded by the *var* multigene family have been suggested as possible GSA 10. Transcripts of *var* genes have been found in late stage gametocytes 22 and more recently, PfEMP1 has been detected on the surface of early maturing gametocytes but not in knobs associated with adhesion as is the case with asexual parasites 14. These minimum levels of PfEMP1 expression on the surface of immature gametocytes do not support adhesive interactions with endothelial receptors and studies of *var* gene transcription revealed a decline in transcript abundance in the later stages of gametocyte development 14, 22. The RIFIN and STEVOR proteins are also encoded by subtelomeric multigene families, and are expressed in later stage gametocytes. Immunofluorescence studies localized these proteins to the plasma membrane of gametocyte‐infected erythrocytes, but surface exposure has not as yet been demonstrated 21, 42. Further, the disappearance of STEVOR proteins is correlated with deformability of gametocytes as they mature, with less detectable STEVOR proteins in most mature deformable stage V gametocytes 13. Functional assays are required to test empirically whether STEVOR, Rifins and/or PfEMP1 contribute to the surface of mature gametocyte‐infected erythrocytes, or whether GSA, which our data suggest are heterogeneous molecules and only transiently expressed, are encoded by yet unidentified genes. A member of the CPW‐WPC gene family, previously suggested as a putative GSA 12, is now known to be expressed in ookinetes 50, and thus unlikely to be exposed on the surface of gametocyte‐infected erythrocytes.

## Conclusions

This study provides the first evidence of *P. falciparum* gametocyte‐specific antibody responses in asymptomatic children, and showed that anti‐GSA antibodies from Ghanaian children were able to recognize GSA of a clinical isolate from Kenya. Anti‐GSA antibodies were found to be weakly associated with lower risk of gametocyte development 3–4 weeks after antibody detection. The identification of the targets of these antibodies is now an important goal, which would enable exploration of GSA as candidate transmission‐blocking vaccine targets, and of anti‐GSA IgG as a biomarker of gametocyte carriage.

## Author contributions

B.D., C.J.S. and G.T. conceived and designed the study. B.D. and E.K. carried out the experiments and C.J.S and B.D. analysed the data. B.D. wrote the manuscript with contributions from C.J.S., E.K. and G.T. All authors approved the final manuscript.
